# Effects of maternal pre-pregnancy body mass index and gestational weight gain on antenatal mental disorders in China: a prospective study

**DOI:** 10.1186/s12884-023-05502-y

**Published:** 2023-03-18

**Authors:** Xuan Zhou, Lin Rao, Dongjian Yang, Tong Wang, Hong Li, Zhiwei Liu

**Affiliations:** 1 School of Medicine, The International Peace Maternity and Child Health Hospital, Shanghai Jiao Tong University, 910 Hengshan Road, Xuhui District, 200030 Shanghai, China; 2 Shanghai Key Laboratory of Embryo Original Diseases, 200030 Shanghai, China; 3 Institute of Birth Defects and Rare Diseases, School of Medicine, Shanghai Jiao Tong University, 200030 Shanghai, China; 4 Shanghai Jiao Tong University School of Medicine, 200030 Shanghai, China

**Keywords:** Pregnancy, Gestational weight gain, Antenatal mental disorders, Depression, Anxiety, Stress

## Abstract

**Background:**

Maternal obesity is the most common medical condition among women of reproductive age worldwide. The pre-pregnancy body mass index and gestational weight gain have been suggested to be associated with maternal mental disorders. This study aimed to investigate the effects of the pre-pregnancy body mass index and gestational weight gain on antenatal depression, stress, and anxiety.

**Methods:**

In total, 4,890 pregnant women were enrolled in the present study, which is based on an ongoing prospective cohort study. We used self-reported pre-pregnancy weights and the last weights measured prior to delivery (using professional instruments) to calculate the pre-pregnancy body mass index and gestational weight gain. The questionnaires used included the Center for Epidemiologic Studies Depression Scale (CES-D), Self-Rating Anxiety Scale (SAS), and 10-item version of the Perceived Stress Scale (PSS-10). We used Pearson product-moment correlation and multivariable logistic regression models to examine the impact of the pre-pregnancy body mass index and gestational weight gain on different maternal mental disorders.

**Results:**

After adjusting for conception, annual household income, occupation, education, smoking status, and drinking status, excessive gestational weight gain during pregnancy was associated with a greater chance of anxiety symptoms in the entire sample (adjusted model: odds ratio = 1.479, 95% confidence interval = 1.128, 1.938) and especially in women with a normal body mass index (adjusted model: odds ratio = 1.668, 95% confidence interval = 1.209, 2.302). However, the relationship between the maternal pre-pregnancy body mass index and mental health was not significant.

**Conclusion:**

Pregnant women with a normal pre-pregnancy body mass index had a greater chance of experiencing anxiety symptoms before delivery if gestational weight gain was excessive; however, its effects on depression or stress symptoms were not observed. The maternal pre-pregnancy body mass index may not be independently associated with maternal mental disorders.

## Background

Pregnancy is a period of significant neurobiological and psychological changes brought about by physiological hormones, and is usually accompanied by an increase in various negative emotions [1]. Approximately one in five women experience antenatal mental disorders, such as depression and anxiety [2]. Perinatal depression (period prevalence: 18% for prenatal depression and 14% for postpartum depression) is one of the most common psychological problems in pregnant women [3]; its prevalence may be even higher in Asian populations (24.3%) [4]. Perinatal anxiety and stress affect approximately 17% and up to 84% of all women, respectively [5]. A Chinese study revealed that more than 50% of pregnant women have symptoms of anxiety and stress, especially in the late pregnancy and postpartum periods [6].Poor dietary intake and social support, an increased risk of preeclampsia, and pregnancy and labour complications are some harmful consequences of anxiety, depression, and stress during the antenatal period [7]. Furthermore, maternal mental disorders are associated with several adverse foetal and neonatal outcomes, such as low birth weights, preterm births, high rates of diarrhoea, poor breastfeeding practices, infectious illnesses, and poor cognitive development [8].

Maternal obesity is a growing public health concern; in fact, it is the most common medical condition in women of reproductive age worldwide [9].Women who are overweight or obese are more likely to experience excessive gestational weight gain (GWG) and postpartum weight retention [10]. The prevalence of obesity during pregnancy differs according to diverse guidelines [11]. Studies in population-based cohorts have shown that significant ethnic differences in the genetic background, living environment, and lifestyle lead to population-level differences in the body mass index (BMI) and GWG [12]. For instance, according to traditional Chinese customs, pregnant women are frequently required to overeat certain foods and reduce exercise during pregnancy [13]; however, overnutrition and a lack of exercise in mothers may lead to excessive GWG, postpartum weight retention, and macrosomia [14].

Evidence suggests that pre-pregnancy obesity and excessive GWG may increase the risk of adverse mental health outcomes in pregnant women [15–17]. Moreover, these factors may also negatively impact a woman’s self-image and self-esteem [18]. Ertel et al. found that pre-pregnancy obesity was associated with elevated depressive symptoms during the postpartum period [19]. A systematic review and meta-analysis also suggested that pre-pregnancy obesity was associated with an increased risk of maternal depressive symptoms and anxiety during pregnancy and in the postpartum period [17]. Kominiarek et al. conducted a survey to evaluate the association between prenatal stress and GWG; their findings suggested that the lowest stress scores were associated with adequate GWG [20]. However, all of these studies have considered a single symptom to measure the mental health of pregnant mothers and have ignored the multiple negative emotions experienced by these women. Stress, anxiety, and depressive symptoms are intercorrelated; their active assessment and management is required in the perinatal period [6].

Understanding the risk factors of perinatal emotion disorders, especially those that can be modified, can improve our ability to identify women at risk and provide additional ways for possible prevention and intervention. To the best of our knowledge, there are few studies (especially those considering racial factors) on the effects of pre-pregnancy BMI and GWG on antenatal mental disorders. In the present study, we aimed to assess the prevalence of pre-pregnancy BMI and GWG in and the sociodemographic characteristics of pregnant Chinese women. We hypothesized that the pre-pregnancy BMI and GWG would predict depression, stress, and/or anxiety symptoms during pregnancy.

## Methods

### Design and participants

This prospective cohort study was performed as part of the ongoing China National Birth Cohort Study from March 2017 to May 2020. The study design was approved by the Ethics Committee of the International Peace Maternity and Child Health Hospital (affiliated with the Shanghai Jiao Tong University School of Medicine; approval no.: GKLW2016-21).Study participation was voluntary; the patients declared their willingness to participate only after examining the objectives and procedures of the study. All enrolled patients provided written informed consent.

The inclusion criteria were as follows: (i) women planning to seek prenatal care and deliver at the study hospital, (ii) women whose maternity files were established in the hospital, (iii) women with singleton pregnancies, (iv) women who could complete online questionnaires in Chinese, and (v) women willing to sign the consent form. The exclusion criteria were as follows: (i) women aged < 20 years; (ii) women with mental disorders, including depression or anxiety before pregnancy; (iii) women with mental disorders in the first and second trimesters of pregnancy; and (iv) women with serious underlying conditions. After enrolment, structured questionnaires were administered to pregnant women hospitalised for childbirth or before delivery. Thereafter, women with preterm births (i.e., delivery before 37 completed weeks of gestation); those who transferred to another hospital; those with insufficient pre-pregnancy BMI, weight, and height data; and those with other missing data were excluded. The final study sample comprised 4,890 women.

### Measurements

#### BMI and GWG

BMI is a statistical index that is computed using a person’s weight and height; it provides an estimate of the body fat in men and women of any age. Pre-pregnancy BMI was calculated by dividing the participants’ pre-pregnancy weight (in kg) by their height (in m^2^). Based on the adult BMI classification standards for the Chinese population [21], we categorized our participants into the following four groups using their BMI: underweight (BMI < 18.5 kg/m^2^), normal weight (18.5 kg/m^2^ ≤ BMI < 24 kg/m^2^), overweight (24 kg/m^2^ ≤ BMI < 28 kg/m^2^), and obese (BMI ≥ 28 kg/m^2^).

GWG data were obtained by computing the difference between the last weight measured using professional instruments before delivery in the hospital and the self-reported pre-pregnancy weight. However, there are no official guidelines on GWG for the Chinese population. In the current study, maternal GWG was defined as excessive, adequate, and insufficient. We adopted the Institute of Medicine’s (IOM) recommendation for GWG and defined adequate GWG as being 12.5–18.0 kg, 11.5–16.0 kg, 7.0–11.50 kg, and 5.0–9.0 kg in underweight women, women with normal weight, overweight women, and obese women, respectively [22].

#### Measurement of depressive symptoms

The Center for Epidemiologic Studies Depression Scale (CES-D) was used to measure the level of depressive symptoms in the enrolled women. The CES-D is a 20-item tool, with each item rated on a 4-point scoring system; the total score ranges from 0 to 60. Higher scores indicated a higher probability of an individual experiencing depression. A cut-off score of 16 was used to determine non-depression/depression in pregnant women and postpartum mothers [23]. The Chinese version of the scale has been reported to have good reliability and validity [24]. The Cronbach’s α was 0.924 in the current study.

#### Measurement of anxiety symptoms

Anxiety symptoms were assessed using the Self-Rating Anxiety Scale (SAS), which was developed in 1971. It is primarily used to evaluate the severity of an individual’s anxiety. It is a 20-item scale, with each item rated using a 4-level score [25]. Higher scores indicate more severe anxiety symptoms.SAS has been reported to have good reliability and validity in China, and a standardised score of 50 is the upper limit for normative populations [26]. The Cronbach’s α was 0.894 in the current study.

#### Measurement of perceived stress

Perceived stress was assessed using the 10-item Perceived Stress Scale (PSS-10). Each item was scored on a 5-point scale, with the total score ranging from 0 to 40 [27]. Higher scores indicate higher levels of perceived stress, and scores of 14 or above are indicative of moderate-to-high levels of perceived stress [28]. The Chinese version of this scale has demonstrated good reliability and validity [29]. The Cronbach’s α was 0.833 in the current study.

#### Assessment of covariates

Several variables were assessed, including age, gestational age at delivery, method of conception, education, occupation, annual household income, parity, place of residence, ethnicity, drinking status, and smoking status. Sociodemographic data were assessed using an interviewer-administered questionnaire before delivery. If the gestational week differed from the delivery gestational week, the researcher manually modified it according to medical records.

### Statistical analysis

Descriptive statistics were used to summarise the participants’ characteristics. Continuous variables are expressed as means ± standard deviations, whereas categorical variables are expressed as percentages. An analysis of variance was performed to analyse the differences among the groups. The Chi-square and Fisher exact tests were performed to examine the association between two categorical variables. A Pearson’s correlation coefficient analysis was undertaken as an exploratory analysis to explore the relationship among the pre-pregnancy BMI, GWG, CES-D score, and SAS score. A binary logistic regression was performed to calculate the odds ratios (ORs) and 95% confidence intervals (CIs) for the relationships among the pre-pregnancy BMI, GWG, and risk of exceeding scale thresholds (CES-D, SAS, and PSS-10 scores) in the entire sample. All statistical analyses were two-sided and performed using R version 4.0.5 (R Foundation for Statistical Computing, Vienna, Austria); *p* < 0.05 indicated statistical significance.

## Results

Among 4,890 participants, 999 (20.4%), 2,059 (42.1%), and 1,832 (37.7%) had insufficient, adequate, and excessive GWG, respectively. Furthermore, 53.5% and 43.4% of the participants with an underweight and a normal BMI before pregnancy, respectively, had adequate GWG; conversely, 64.0% and 73.2% of the participants with an overweight and obese BMI before pregnancy had excessive GWG, respectively. Table 1 presents the participants’ demographic data according to the pre-pregnancy BMI categories. No significant differences were observed among the four groups in terms of the place of residence, occupation, ethnicity, smoking status, and drinking status (*p* > 0.05 for all). Women with pre-pregnancy obesity tended to be younger and had a higher proportion of excessive GWG (all *p* < 0.05). Significant intergroup differences were observed in terms of conception, annual household income, education, and parity: most women who conceived naturally and were underweight were more likely to have relatively a higher income and education level, and most were nulliparous before this pregnancy.

**Table 1:** Characteristics of participants by pre-pregnancy BMI

Variables	Total (*n* = 4890)	Pre-BMI underweight (*n* = 665)	Pre-BMI normal (*n* = 3474)	Pre-BMI overweight (*n* = 639)	Pre-BMI obesity (*n* = 112)	*F/χ* ^*2*^	*p*
Age, Mean ± SD	30.59 ± 3.57	31.94 ± 3.88	32.65 ± 4.10	32.71 ± 4.23	30.59 ± 3.57	34.848	< 0.001
Gestational weight gain, Mean ± SD	39.21 ± 0.99	39.26 ± 1.02	39.19 ± 0.98	38.89 ± 0.88	39.21 ± 0.99	5.282	0.001
Gestational weight gain, *n* (%)						382.345	< 0.001
Insufficient	999 (20.4)	194 (29.2)	741 (21.3)	58 (9.1)	6 (5.4)		
Adequate	2059 (42.1)	356 (53.5)	1507 (43.4)	172 (26.9)	24 (21.4)		
Excessive	1832 (37.5)	115 (17.3)	1226 (35.3)	409 (64)	82 (73.2)		
Method of conception, *n* (%)						30.376	< 0.001
Assisted reproductive technology	859 (17.6)	72 (10.8)	627 (18)	141 (22.1)	19 (17)		
Conceived Naturally	4031 (82.4)	593 (89.2)	2847 (82)	498 (77.9)	93 (83)		
Place of residence, *n* (%)						-	0.405
Rural	218 (4.5)	38 (5.7)	148 (4.3)	27 (4.2)	5 (4.5)		
Urban	4672 (95.5)	627 (94.3)	3326 (95.7)	612 (95.8)	107 (95.5)		
Annual household income (10,000 RMB (1410 USD)/year), *n* (%)						44.903	< 0.001
~10	1542 (31.5)	205 (30.8)	1028 (29.6)	259 (40.5)	50 (44.6)		
20 ~ 30	1491 (30.5)	191 (28.7)	1082 (31.1)	190 (29.7)	28 (25)		
30~	1857 (38)	269 (40.5)	1364 (39.3)	190 (29.7)	34 (30.4)		
Occupation, *n* (%)						1.963	0.580
Employed	4376 (89.5)	591 (88.9)	3122 (89.9)	564 (88.3)	99 (88.4)		
Unemployed	514 (10.5)	74 (11.1)	352 (10.1)	75 (11.7)	13 (11.6)		
Education, *n* (%)						35.197	< 0.001
~College/vocational	1187 (24.3)	138 (20.8)	821 (23.6)	189 (29.6)	39 (34.8)		
Undergraduate	2566 (52.5)	382 (57.4)	1793 (51.6)	334 (52.3)	57 (50.9)		
Postgraduate~	1137 (23.3)	145 (21.8)	860 (24.8)	116 (18.2)	16 (14.3)		
Parity, *n* (%)						19.191	< 0.001
Nulliparous	3976 (81.3)	581 (87.4)	2799 (80.6)	507 (79.3)	89 (79.5)		
Multiparous	914 (18.7)	84 (12.6)	675 (19.4)	132 (20.7)	23 (20.5)		
Ethnic, *n* (%)						-	0.858
Han	4797 (98.1)	655 (98.5)	3404 (98)	628 (98.3)	110 (98.2)		
non-Han	93 (1.9)	10 (1.5)	70 (2)	11 (1.7)	2 (1.8)		
^a^ Smoking status, *n* (%)						-	0.110
No	4772 (97.7)	642 (96.5)	3400 (98)	622 (97.5)	108 (97.3)		
Yes	111 (2.3)	23 (3.5)	69 (2)	16 (2.5)	3 (2.7)		
^b^ Drinking status, *n* (%)						3.927	0.270
No	2999 (61.4)	390 (58.6)	2141 (61.7)	404 (63.3)	64 (57.7)		
Yes	1884 (38.6)	275 (41.4)	1328 (38.3)	234 (36.7)	47 (42.3)		

**Abbreviations**: *SD* standard deviation, *BMI* body mass index, *GWG* gestational weight gain

^a^ Smoking during pregnancy or during the year before pregnancy. ^b^ Alcohol consumption during pregnancy or during the year before pregnancy

Table 2 shows a comparison of the scale scores (CES-D, SAS, and PSS-10 scores) among different GWG subgroups according to the total sample and the pre-pregnancy BMI categories. In the total sample and among normal-weight women, the SAS scores were higher in those with excessive GWG than in those with insufficient or adequate GWG (*p* < 0.001). No such differences were observed among the GWG subgroups for the other pre-pregnancy BMI categories. Furthermore, 7.8% and 7.2% of the patients with normal pre-pregnancy BMI and in the total sample, respectively, had SAS scores ≥ 50. Among women with a normal BMI, the PSS-10 score was higher in those with excessive GWG than in those with insufficient and adequate GWG. No such differences were observed among the GWG subgroups for the other pre-pregnancy BMI categories. Furthermore, the likelihood of the PSS-10 score being ≥ 14 was higher in both the total sample and in women with a normal BMI than in the women from the other BMI categories. Conversely, the CES-D score did not differ significantly among the GWG subgroups in all the pre-pregnancy BMI categories (all *p* > 0.05).

**Table 2:** Scale scores of pregnant women in subgroups of GWG by total sample and pre-pregnancy BMI

Variables	*n*	GWG insufficient	GWG adequate	GWG excessive	*p*
**Total**	4890				
CES-D score		8.90 ± 7.50	9.20 ± 7.63	9.40 ± 8.07	0.251
< 16		807 (80.8)	1647 (80.0)	1444 (78.8)	0.427
≥ 16		192 (19.2)	412 (20.0)	388 (21.2)	
SAS score		35.78 ± 7.06	35.97 ± 6.74	36.85 ± 7.44	< 0.001
< 50		943 (94.4)	1960 (95.2)	1700 (92.8)	0.006
≥ 50		56 (5.6)	99 (4.8)	132 (7.2)	
PSS-10 score		9.44 ± 5.54	9.73 ± 5.47	9.95 ± 5.79	0.070
< 14		780 (78.1)	1562 (75.9)	1346 (73.5)	0.020
≥ 14		219 (21.9)	497 (24.1)	486 (26.5)	
**Pre-BMI normal**	3474				
CES-D score		8.73 ± 7.53	9.15 ± 7.65	9.60 ± 8.09	0.052
< 16		605 (81.6)	1212 (80.4)	953 (77.7)	0.076
≥ 16		136 (18.4)	295 (19.6)	273 (22.3)	
SAS score		35.62 ± 7.10	35.79 ± 6.68	36.94 ± 7.66	< 0.001
< 50		700 (94.5)	1438 (95.4)	1130 (92.2)	0.001
≥ 50		41 (5.5)	69 (4.6)	96 (7.8)	
PSS-10 score		9.31 ± 5.57	9.66 ± 5.43	10.06 ± 5.79	0.015
< 14		586 (79.1)	1151 (76.4)	888 (72.4)	0.002
≥ 14		155 (20.9)	356 (23.6)	338 (27.6)	
**Pre-BMI underweight**	665				
CES-D score		9.68 ± 7.38	9.72 ± 7.45	9.06 ± 7.72	0.716
< 16		148 (76.3)	276 (77.5)	92 (80.0)	0.751
≥ 16		46 (23.7)	80 (22.5)	23 (20.0)	
SAS score		36.52 ± 6.88	36.84 ± 6.95	36.77 ± 7.25	0.871
< 50		184 (94.8)	335 (94.1)	108 (93.9)	0.921
≥ 50		10 (5.2)	21 (5.9)	7 (6.1)	
PSS-10 score		9.61 ± 5.58	10.26 ± 5.42	9.73 ± 6.19	0.370
< 14		143 (73.7)	260 (73.0)	84 (73.0)	0.984
≥ 14		51 (26.3)	96 (27.0)	31 (27.0)	
**Pre-BMI overweight**	639				
CES-D score		8.72 ± 7.52	8.89 ± 7.76	9.08 ± 8.14	0.928
< 16		49 (84.5)	138 (80.2)	331 (80.9)	0.770
≥ 16		9 (15.5)	34 (19.8)	78 (19.1)	
SAS score		35.10 ± 7.10	36.10 ± 6.66	36.79 ± 7.22	0.181
< 50		53 (91.4)	164 (95.3)	382 (93.4)	0.472
≥ 50		5 (8.6)	8 (4.7)	27 (6.6)	
PSS-10 score		10.79 ± 4.99	9.62 ± 5.59	9.71 ± 5.66	0.281
< 14		45 (77.6)	130 (75.6)	311 (76.0)	0.953
≥ 14		13 (22.4)	42 (24.4)	98 (24.0)	
**Pre-BMI obesity**	112				
CES-D score		6.50 ± 5.79	7.29 ± 7.82	8.59 ± 7.78	0.621
< 16		5 (83.3)	21 (87.5)	68 (82.9)	0.900
≥ 16		1 (16.7)	3 (12.5)	14 (17.1)	
SAS score		37.33 ± 6.83	33.50 ± 6.47	35.83 ± 5.26	0.274
< 50		6 (100.0)	23 (95.8)	80 (97.6)	0.611
≥ 50		0 (0.0)	1 (4.2)	2 (2.4)	
PSS-10 score		6.67 ± 4.18	7.04 ± 6.84	9.80 ± 5.86	0.112
< 14		6 (100.0)	21 (87.5)	63 (76.8)	0.308
≥ 14		0 (0.0)	3 (12.5)	19 (23.2)	

**Abbreviations**: *CES-D* Center for Epidemiologic Studies Depression Scale, *SAS* Self-Rating Anxiety Scale, *PSS-10* 10-item version of Perceived Stress Scale, *BMI* body mass index, *GWG* gestational weight gain

**Fig. 1:**
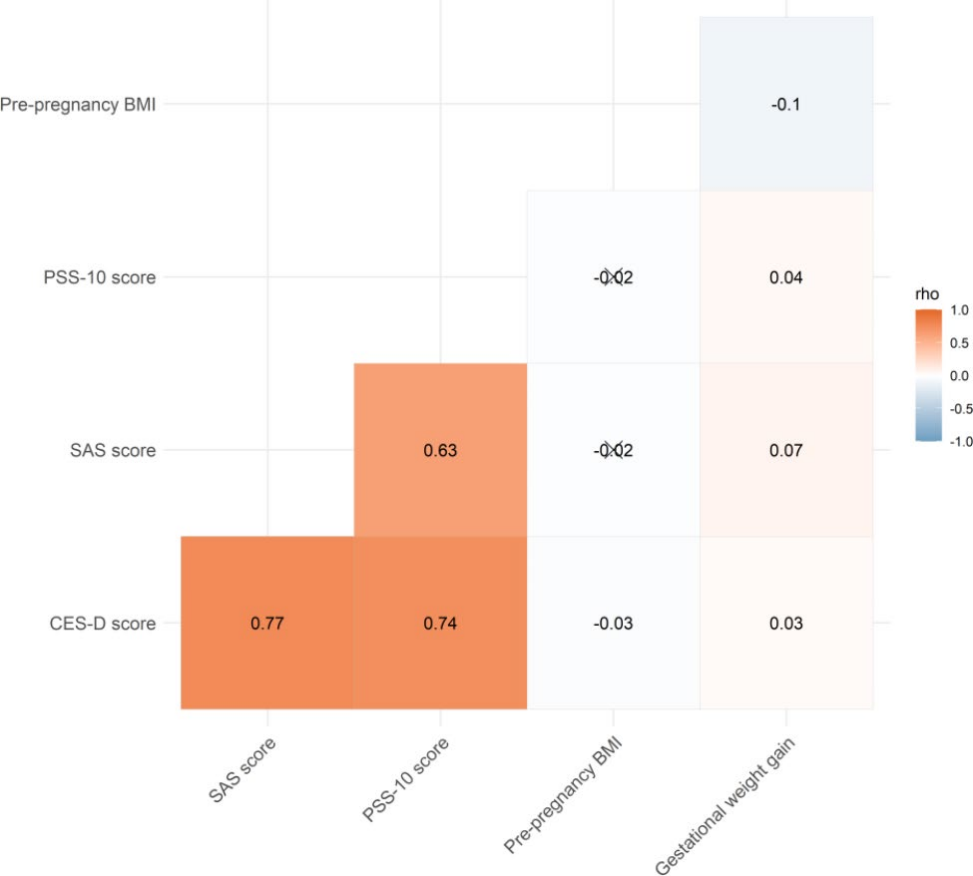
Correlation heat map of pre-pregnancy BMI, GWG, and the three scale scores

Low correlations were observed among the pre-pregnancy BMI, GWG, and the three scale scores. No significant association was found among the PSS-10 score, SAS score, and pre-pregnancy BMI (all *p* > 0.05). However, a strong correlation was found between the CES-D and SAS scores (rho = 0.77, *p* < 0.05), CES-D and PSS-10 scores (rho = 0.74, *p* < 0.05), and SAS and PSS-10 scores (rho = 0.63, *p* < 0.05).This indicates that the three scale scores were positively correlated with each other (See Fig. 1 for details).

A logistic regression analysis was performed to explore the relationship between GWG and maternal mental disorders (Table 3). Excessive GWG was found to be associated with exceeding the SAS score threshold in the entire sample (OR = 1.535; 95% CI = 1.173, 2.008). After adjusting for potential confounders, excessive GWG was also found to be associated with a greater chance of anxiety symptoms in the entire sample (OR = 1.479; 95% CI = 1.128, 1.938).

Another logistic regression analysis was performed to explore the relationship between GWG and depression, anxiety, and stress according to the pre-pregnancy BMI. Excessive GWG significantly increased the possibilities of anxiety in both the unadjusted and adjusted models (unadjusted model: OR = 1.767, 95% CI = 1.284, 2.431; adjusted model: OR = 1.668, 95% CI = 1.209, 2.302). Conversely, excessive GWG was significantly associated with the chance of exceeding the PSS-10 score thresholds in the unadjusted model (OR = 1.227; 95% CI = 1.033, 1.459) but not in the adjusted model (OR = 1.151; 95% CI = 0.966, 1.372).

**Table 3:** Associations among maternal pre-pregnancy BMI, GWG and the exceeding scale thresholds in total sample

Variables	CES-D				SAS				PSS-10		
	GWG insufficient	GWG adequate	GWG excessive		GWG Insufficient	GWG adequate	GWG excessive		GWG insufficient	GWG adequate	GWG excessive
**Total**											
Model 1^a^	0.951 (0.785,1.151)	Reference	1.075 (0.92,1.257)		1.179 (0.842,1.651)	Reference	**1.535 (1.173,2.008)**		0.88 (0.734,1.055)	Reference	1.133 (0.98,1.309)
Model 2^b^	0.961 (0.793,1.164)	Reference	1.052 (0.899,1.231)		1.2 (0.855,1.683)	Reference	**1.479 (1.128,1.938)**		0.905 (0.754,1.087)	Reference	1.073 (0.927,1.243)
**Pre-BMI normal**											
Model 1	0.928 (0.741,1.163)	Reference	1.179 (0.98,1.419)		1.222 (0.821,1.817)	Reference	**1.767 (1.284,2.431)**		0.849 (0.686,1.051)	Reference	**1.227 (1.033,1.459)**
Model 2	0.939 (0.748,1.177)	Reference	1.14 (0.946,1.374)		1.248 (0.838,1.86)	Reference	**1.668 (1.209,2.302)**		0.875 (0.705,1.086)	Reference	1.151 (0.966,1.372)
**Pre-BMI underweight**											
Model 1	1.072 (0.709,1.622)	Reference	0.862 (0.513,1.451)		0.867 (0.4,1.88)	Reference	1.034 (0.428,2.499)		0.966 (0.65,1.436)	Reference	0.9995 (0.6224,1.6052)
Model 2	1.083 (0.709,1.655)	Reference	0.836 (0.493,1.418)		0.85 (0.386,1.871)	Reference	1.055 (0.433,2.571)		0.969 (0.644,1.459)	Reference	0.954 (0.588,1.548)
**Pre-BMI overweight**											
Model 1	0.663 (0.287,1.529)	Reference	0.956 (0.61,1.499)		1.971 (0.618,6.288)	Reference	1.449 (0.645,3.257)		0.915 (0.45,1.86)	Reference	0.975 (0.644,1.478)
Model 2	0.687 (0.293,1.612)	Reference	0.946 (0.6,1.49)		2.145 (0.658,6.993)	Reference	1.459 (0.646,3.294)		0.919 (0.445,1.899)	Reference	0.942 (0.619,1.435)
**Pre-BMI obesity**											
Model 1	1.75 (0.143,21.384)	Reference	1.441 (0.378,5.501)		0 (0,Inf)	Reference	0.575 (0.05,6.629)		0 (0,Inf)	Reference	2.111 (0.567,7.855)
Model 2	2.829 (0.162,49.469)	Reference	1.368 (0.324,5.784)		0 (0,Inf)	Reference	0.229 (0.014,3.746)		0 (0,Inf)	Reference	1.504 (0.379,5.958)

**Abbreviations**:﻿ CES-D, Center for Epidemiologic Studies Depression Scale; SAS, Self-Rating Anxiety Scale;PSS-10, 10-item version of Perceived Stress Scale; BMI, body mass index; GWG, gestational weight gain

^a^: Model 1 unadjusted logistic regression model

^b^: Model 2 was adjusted for age at conception, annual household income, occupation, education, smoking status, and drinking status

## Discussion

The main purpose of our study was to explore the impact of pre-pregnancy BMI and GWG on the psychological state of women. The most important finding of this study is that excessive GWG is strongly associated with greater chances of exceeding the SAS score thresholds in pregnant Chinese women with a normal pre-pregnancy BMI, while in no other category were the results significant.No effects of GWG on depression or stress symptoms were observed. In other words, pregnant women with normal weight before pregnancy are more likely to have anxiety symptoms if they gain too much weight during pregnancy. We did not find a strong association between the pre-pregnancy BMI and the perinatal psychological status. These findings suggest that avoiding excessive GWG can reduce the incidence of anxiety in pregnant women with a normal pre-pregnancy weight.

In the present study, excessive GWG did not affect the women’s stress and depression during pregnancy but aggravated their anxiety. Currently, the conclusions of various relevant studies are not consistent, which indicates that the relationship between GWG and prenatal anxiety, depression, and stress is complex and variable. Ertel et al. did not observe an association between GWG and prenatal depressive symptoms [19]. A study of 505 pregnant women showed that excessive gestational weight gain independently predicted greater postpartum depressive symptoms [16]. In a study focusing on the relationship between stress and GWG, the researchers believed that there might be evidence of an association between stress and maternal body weight and weight gain [30]. Eichler et al. found that GWG was significantly positively linked to stress only during the second trimester [31].The inconsistency in the reported findings can be explained by the following: (1) most of the evidence is based on self-reported body weight and height, which is associated with a higher potential for misclassification (when compared with prospective measurement of maternal BMI) [32] and (2) as a limitation of most studies, the analysed women underestimated their pre-pregnancy weight and overestimated their GWG [33].

Nevertheless, in our study, the negative effect of excessive GWG on perinatal anxiety could not be ignored. We found that women with normal pre-pregnancy weight in our study were at a greater chance of experiencing anxiety symptoms due to excessive GWG. The number of studies on anxiety and weight gain is rather small; however, the findings of some studies are consistent with our findings. Systematic literature suggests that obese pregnant women are at a higher risk of developing comorbid anxiety disorders [34]. Zanardo et al. indicate that women who experienced excessive GWG have a higher risk of developing anxiety [35]. In our study, the finding relates only to women who had normal pre-pregnancy nutritional status. A possible explanation is that women with normal weight before pregnancy receive less relevant education than women with substandard pre-pregnancy weight do; adequate health consultation can positively impact the lifestyle and dietary structure of pregnant women [36]. As healthcare providers tend to focus more on women with obesity [37], women with a normal pre-pregnancy weight may not be able to control their weight correctly in the absence of proper guidance. It is conceivable that women entering pregnancy at an underweight or normal-weight BMI may find the associated changes in body shape more difficult to accept [38]. Studies showed body dissatisfaction might have negative outcomes, including anxiety or feeling stressed, poor self-esteem, isolation, and social anxiety [39, 40]. Another explanation of how GWG affects maternal mental health is inflammatory markers. The study indicated that excessive GWG was associated with higher concentrations of inflammatory factors [41], and inflammation has been implicated in anxiety [42]. Thus, while paying attention to obese or overweight pregnant women, we should also provide adequate advice and health guidance to women with normal pre-pregnancy weights.

At the same time, it should be noted that the incidence of perinatal anxiety was relatively low in our study as compared to in previous studies; these previous studies have revealed that 15–30% of pregnant women have clinically significant levels of anxiety [43, 44]. With an SAS score of 50 as the cut-off, only 5.9% of the women were diagnosed with anxiety. Furthermore, 7.8% of women with a normal pre-pregnancy weight and excessive GWG were diagnosed with anxiety symptoms. The large difference in the incidence may also explain the inconsistencies in the reported effects of GWG on prenatal anxiety among different studies.

Our hypothesis that pre-pregnancy BMI was associated with depression, stress, and/or anxiety symptoms during pregnancy was not supported by our data; this is consistent with the findings of other studies. McPhie et al. reported that pre-pregnancy BMI could not predict depressive or anxiety symptoms [45]. However, there are conflicting findings regarding pre-pregnancy and antenatal mental disorders. A systematic review and meta-analysis of the impact of pre-pregnancy BMI on maternal depressive and anxiety symptoms revealed positive associations between pre-pregnancy weight and depression and anxiety [17]. Another study evaluating the prevalence and risk of antenatal mental disorders among obese and overweight women claimed that women who were obese when they became pregnant were more likely to experience elevated antenatal mental health problems [46].

To date, existing findings on the relationship between BMI and mental disorders during pregnancy are inconsistent, and the causal direction of the relationship between obesity and mental health problems in pregnant women is unclear. This inconsistency may be related to the differences in the methodologies adopted (including evaluation instruments and definitions) [47] and to the differences in the adjustments for confounding factors across the studies. In our study, the method of conception, annual household income, occupation, parity, and education were important confounding factors; 68.5% of the participants’ families had a higher annual income, 75.8% of the participants had a bachelor’s degree at the very least, and only 10.5% of the participants were unemployed. In a similar study from Australia, only 61.3% of the women had a Bachelor’s or Master’s degree [47], while Cheng et al. reported that 25% of their participants were unemployed [6]. A community-based cross-sectional study in Aligarh also found that the prevalence of maternal mental disorders was significantly higher among mothers who were part of a higher age group, who belonged to a low socioeconomic class, who had no educational background or had a lower level of education, who were housewives, and who had higher parity [48]. Moreover, social and cultural factors may also affect the body image, weight satisfaction, and weight-gain attitudes of the women [19].

In the present study, only 41.93% of the women met the IOM recommendations and gained an adequate gestational weight; however, as the pre-pregnancy BMI increased, this proportion decreased gradually and the proportion of excessive GWG increased gradually. It is particularly concerning that current evidence shows that up to 64% of overweight women and 73.2% of obese women have GWG that exceeds the GWG currently recommended by IOM; this is consistent with our findings [49, 50]. Healthcare providers should calculate the pre-pregnancy BMI of pregnant women at their first prenatal visit, so that they can provide optimal diet and exercise counselling for achieving the IOM-recommended GWG.

We also noticed positive correlations among the perceptions of depression, anxiety, and stress. Previous studies have proposed that perceived stress, anxiety, and depression are strongly correlated with pregnancy [51, 52]. Several comorbidities may exist among maternal mental disorders. Dindo et al. found high rates of co-occurrence of anxiety and stress disorders in women with depression [53]. Because of this increasing emphasis on screening for comorbidities during pregnancy, it is necessary to further study depression, stress, and anxiety as comorbidities as well.

To the best of our knowledge, this study included the largest sample to date to explore the relationship among the pre-pregnancy BMI, GWG, and maternal mental health in the Chinese population. However, our study has some limitations. First,although the weight during pregnancy was measured using professional measuring machines, the pre-pregnancy weight was self-reported; this may have led to some potential misclassification. Second, some major complications, such as gestational diabetes mellitus, hypertension disorders, and thyroid dysfunction, were not evaluated in our study, even though these are important confounders in the evaluation of maternal mental disorders. Finally, assessments of mental health were derived from self-reported questionnaires, which may be susceptible to measurement bias.

## Conclusion

This large prospective cohort study demonstrated that women with normal weight before pregnancy are more likely to have anxiety symptoms if they gain too much weight during pregnancy. However, our findings did not indicate a strong association between the pre-pregnancy BMI and the perinatal psychological status. Instead, our findings suggest that excessive GWG is associated with antenatal anxiety,in women with a normal pre-pregnancy weight. Thus, such women should receive sufficient attention and health guidance, because poor weight control during pregnancy might aggravate their anxiety symptoms. More than 50% of the women in our study did not meet the IOM standards for adequate GWG. In the future, larger longitudinal studies are needed to explore the biological mechanisms linking GWG with maternal mental health. Screening and interventions should be performed to identify women at a high risk of excess GWG relatively early in pregnancy to prevent the occurrence of mental disorders.

## Data Availability

The datasets used and/or analysed during the current study are available from the corresponding author on reasonable request.

## Abbreviations

BMI: Body mass index
CES-D: Center for Epidemiologic Studies Depression Scale
CI: Confidence interval
GWG: Gestational weight gain
HPA: Hypothalamic pituitary adrenal
IOM: Institute of Medicine
OR: Odds ratio
PSS-10: 10-item version of the Perceived Stress Scale
SAS: Self-Rating Anxiety Scale
SD: Standard deviation
